# Evaluation of food consumption, energy availability and adherence to the Mediterranean dietary pattern in a group of non-professional athletes

**DOI:** 10.3389/fnut.2026.1858754

**Published:** 2026-07-24

**Authors:** Ellis Bianchi, Massimiliano Tucci, Simone Perna, Daniela Martini, Cristian Del Bo', Alessandro Leone, Francesca Menichetti, Simona Bertoli, Alberto Battezzati, Patrizia Riso

**Affiliations:** 1 Division of Human Nutrition, Department of Food, Environmental and Nutritional Sciences (DeFENS), Università degli Studi di Milano, Milan, Italy; 2 International Center for the Assessment of Nutritional Status and Development of Dietary Intervention Strategies (ICANS-DIS), Department of Food, Environmental and Nutritional Sciences (DeFENS), University of Milan, Milan, Italy

**Keywords:** dietary assessment, energy intake, food consumption assessment, Mediterranean diet, non-professional athletes, sport nutrition

## Abstract

**Background and aim:**

Adequate nutritional intake is fundamental for optimizing athletic performance and maintaining health. The aim of the present study was to investigate the dietary habits of a group of non-professional athletes, assessing their capacity to cover energy, macronutrient, and micronutrient requirements, as well as their adherence to the Mediterranean dietary pattern.

**Methods:**

Sixty-seven participants completed a 7-day food diary to assess nutrient intake and food group consumption. Anthropometric measurements were performed to estimate body composition and Energy Availability (EA). To ensure data accuracy, misreporting was assessed, and only 45 participants classified as “adequate energy reporters” were included in the final analysis.

**Results:**

The mean EA was 33.3 ± 7.0 kcal/kg fat-free mass (FFM), which, while above the threshold for physiological disruption, remains below the optimal level of 45 kcal/kg FFM. In terms of macronutrients, carbohydrate intake (4.7 ± 0.9 g/kg body weight) showed a tendency to not meet the recommended threshold for moderate physical activity (5–8 g/kg). Regarding adherence to the Mediterranean dietary pattern, the mean adherence score was 9.7 ± 2.5, suggesting a generally good adherence; however, food groups consumption analysis revealed a lower than recommended intake of fruits, vegetables, and extra virgin olive oil, while of the consumption of red meat and bakery products such as bread substitutes and sweet baked products, frequently exceeded amounts suggested in the guidelines. This suboptimal diet quality was coupled with insufficient intake of key micronutrients such as vitamin E, folate, magnesium, and potassium.

**Discussion:**

These findings highlight a gap between actual nutritional intake and national dietary reference values in non-professional athletes. The results emphasize the need for targeted nutritional education to address misconceptions about energy and carbohydrate consumption and to prevent potential health risks associated with low energy availability and inadequate diet quality.

## 1 Introduction

It is now well established that adequate diet and energy intake are fundamental in individuals who engage in sports, as it ensures optimal body function, facilitate appropriate macronutrient and micronutrient intake, and contributes to the maintenance of an optimal body composition (1). Indeed, studies consistently demonstrate that insufficient energy intake or an imbalance in macronutrients can compromise not only athletic performance but also metabolic and physiological function (2, 3). Furthermore, following an energy-deficient diet during training can lead to reductions in muscle mass, strength, and bone mineral density, as well as a heightened vulnerability to illness and injury (4). In 2007 the IOC (International Olympic Committee) Consensus group introduced the term “Relative Energy Deficiency in Sport” (RED-S) (5). The primary issue in RED-S is insufficient energy availability (EA) to support the various physiological functions necessary for maintaining optimal health and athletic performance. Healthy physiological function is typically maintained with an EA of 45 kcal per kilogram of fat-free mass per day (kcal/kg FFM/day). However, it has been noticed that many body systems are notably disrupted when EA drops below 30 kcal/kg FFM/day (6). Low EA, due to decreased energy intake or increased exercise demands, leads the body to promote energy rebalance. This can lead to an alteration in hormonal, metabolic, and functional processes (5, 7).

The nutritional needs of athletes are primarily determined by the demands of their activities and their specific objectives and can vary significantly across different populations. Individuals engaging in low levels of physical activity may follow general population guidelines, whereas higher levels of intensity and volume require greater nutritional demands (8). The current literature indicates that athletes and individuals engaged in sports often fail to meet the recommended minimum energy and macronutrient intake, particularly in terms of carbohydrate consumption, however, evidence regarding the nutritional status of this population in Italy remains limited (9–13). Additionally, micronutrient deficiencies have been observed, reflecting a lack of attention to the overall quality of the diet (14).

The Mediterranean dietary pattern is widely recognized as optimal in terms of health outcomes, providing a well-balanced intake of macronutrients and micronutrients (15). The traditional MD is characterized by a high intake of plant-based foods such as fruits, vegetables, and whole grains, with olive oil as the primary fat source. It also includes moderate consumption of dairy, fish, and poultry, while red meat is limited (16). Despite the extensive body of scientific literature, there remains limited evidence on the specific effects of MD for both competitive and amateur athletes (17, 18). Due to its well-balanced intake of nutrients, including carbohydrates making up to 60% of daily energy, the MD pattern can be considered well-suited to meet the nutritional needs of non-professional athletes (19, 20). In this context, the Italian Dietary Guidelines (IDG) provide quantitative standards and suggested frequencies of consumption of key food groups on a daily or weekly basis (21). The guidelines are tailored to different energy requirements and serve as a framework for constructing a balanced diet, based on a Mediterranean dietary pattern.

Beyond the currently available data in literature, it is important to consider that these may not necessarily apply to all different population groups. More specific data are therefore essential to develop dietary recommendations tailored to physically active individuals. Moreover, there is still a gap of knowledge on the actual frequency of consumption of various foods and food groups which is fundamental to understand what athletes typically eat and how to promote necessary adjustments, according to specific guidelines (21). Based on these premises, the aim of the present study was to investigate the dietary habits of a group of non-professional athletes, also to assess whether they met the optimal energy, macronutrients and micronutrients intake. Additionally, an analysis of single foods and overall food categories (i.e., milk and yogurt, cereals, legumes, fish) consumed on a weekly basis was conducted, in order to evaluate the quality of the diet and the adherence to the Mediterranean dietary pattern.

## 2 Materials and methods

### 2.1 Participants recruitment

A total of 84 non-professional athletes and physically active individuals were recruited within the students attending the bachelor's and master's degrees in Sport Sciences and the staff of the University of Milan, as well as via notices posted on bulletin boards and flyers distributed at local sports centers. To be eligible for the study, volunteers were required to be aged between 20 and 45 years, with a Body Mass Index (BMI) ranging from 18 to 30 kg/m^2^. Regarding physical activity habits, participants were required a history of regular training for at least 1 year prior to recruitment, with a total International Physical Activity Questionnaire (IPAQ) questionnaire score over 700 Metabolic Equivalent of Task (MET), corresponding to moderate physical activity level (22).

Exclusion criteria included having any neurological, vascular, or musculoskeletal disorders; taking medications that affect cardiopulmonary or neuromuscular responses; smoking; experiencing menstrual irregularities within 3 months prior to the study; adhering to vegan or vegetarian diets or to any other dietary regimen incompatible with the principles of the Mediterranean dietary pattern; using supplements that could impact the study variables (*e.g.*, creatine, beta-alanine, and protein powder).

This study was carried out in line with the ethical principles reported in the Declaration of Helsinki. All the procedures were approved by Ethics Committee of the University of Milan, Italy (Opinion n. 9/22, 27 January 2022). All participants read and signed a written informed consent form.

### 2.2 Dietary record and analysis

Participants were instructed to complete a 7-day food diary to record information about their habitual dietary habits. Each participant was required to report everything they consumed throughout the day, including the quantity of each food and the recipes for any complex dish. Whenever a meal was consumed outside the home or the exact portion size was unknown, participants were asked to describe the portion as accurately as possible to estimate its quantity. Once participants had completed the food diary, any uncertainties were clarified through a telephone interview. The analysis of the average energy and nutrient intake was performed through Metadieta^®^ software (METEDA srl, Roma, Italy). A comprehensive evaluation could not be performed for certain vitamins and minerals, including vitamin K (phylloquinone and menaquinone), B5 (pantothenic acid), B8 (biotin), copper, iodine, manganese, and selenium, due to significant gaps in the food composition data available in the databases used (*i.e.*, the Food Composition Database for Epidemiological Studies in Italy and the CREA–Council for Agricultural Research and Economics, research center for food and nutrition–food composition database) (23, 24).

A comprehensive analysis of the consumption of various foods and/or food groups was then conducted on a weekly basis, quantifying the total weekly intake and the number of weekly servings. The food groups considered are as follows: milk and yogurt; cheese; butter; pasta; fresh pasta, stuffed pasta and gnocchi; rice; other cereals; bread; potatoes; focaccia, pizza, salad cakes, flatbreads; baked products; breakfast cereals; beef, lamb and pork; cured meat; chicken and other poultry; eggs; fish; canned fish; legumes; nuts; vegetables; fruits; olive oil; other seasoning; jam; fruit juice; biscuits; cakes and brioches; ice cream; other sugars; alcoholic beverages; potato chips.

### 2.3 Assessment of misreporting in food diaries

In the present paper, the ratio between the reported energy intake, estimated through the food diary, and the basal metabolic rate (BMR), calculated using the Schofield equation, was used (25). In the study by Ferraris et al. (26), cut-off values were calculated using the formula outlined by Goldberg et al. (27) to identify the ratio of ‘plausible or adequate energy reporters', considering the entire sample as moderately active. Consequently, subjects with a calculated ratio of EI/BMR within the range of 1.23–2.65 were classified as “plausible” or “adequate energy reporters” (AER). Those with an individual EI < 1.23 were categorized as “under-reporters” or “low energy reporters” (LER), while subjects with an individual EI>2.65 were classified as “over-reporters” (OR). The analyses of energy and nutrient intake, food groups consumed and adherence to the Mediterranean dietary pattern included only diaries categorized as AER, meeting predefined adequacy criteria.

### 2.4 Adherence to Mediterranean diet

Adherence to the Mediterranean dietary pattern was assessed using the validated MEDI-LITE questionnaire (28). The questionnaire was retrospectively completed based on information from 7-day food diaries previously recorded by the volunteers. The questionnaire includes 9 different food groups: fruit, vegetables, legumes, cereals, fish, meat and meat products, dairy products, alcohol, and olive oil. For each food group a sub-score from 0 to 2 was assigned, depending on their alignment with a Mediterranean dietary pattern. The scores for each food diary are then summed, with adherence assessed on a scale from 0 to 18. The higher the final score, the greater the assessed adherence to a MD. According to the MEDI-LITE questionnaire validation study, the optimal cut-off point to discriminate between adherents and non-adherents to a Mediterranean dietary pattern is 8.5 (28).

### 2.5 Anthropometric measurements

Participants underwent anthropometric evaluations following the international guidelines (29), performed by a professional with long-term experience. Subjects had to wear only light underwear during all the evaluations. Body weight was measured using a column scale, and height was measured with a vertical stadiometer; BMI was then calculated as weight (kg) / height (m)^2^. Waist circumference was measured using a non-stretch tape placed midway between the last rib and the iliac crest; hip circumference was measured at the level of the largest lateral extension of the hips. Skinfold thickness (biceps, triceps, subscapular, and suprailiac skinfold) was assessed with a Holtain Tanner/Whitehouse Skinfold Caliper (Holtain Ltd.). Each skinfold was measured three times, and the average of the three readings was used for analysis. Durnin Womersley's formula was used to estimate body density, and body fat percentage was calculated from body density using Siri's formula (30, 31). Fat-free mass (FFM) was subsequently derived by subtracting fat mass from total body weight.

### 2.6 Energy availability calculation

EA refers to the amount of energy remaining for the body's physiological functions after accounting for the energy expended during exercise. EA is calculated as the difference between energy intake (EI) and exercise energy expenditure (EEE), adjusted for FFM (7).


$$\begin{array}{l} EA=\frac{EI\left(kcal\right)-EEE\left(kcal\right)}{FFM\left(kg\right)} \end{array}$$

EI was estimated based on data collected from food diaries, averaging the daily energy intake across seven consecutive days.

EEE was estimated using the IPAQ questionnaire, which participants completed for the same period covered by their food diary (32). This questionnaire allows an estimation of the physical activity performed by the participants throughout the week, quantified as the number of minutes of physical activity, weighted for their intensity expressed as MET. A MET corresponds to the amount of oxygen consumed during rest, equating to approximately 3.5 mL of oxygen per kilogram of body weight per minute (33). For instance, an activity that requires 3 METs means it consumes three times more energy than the resting state. The IPAQ can estimate the total METs for the week by categorizing physical activities into vigorous, moderate, and walking levels, assigning to each a different MET value based on its intensity. An activity with an intensity of 1 MET is conventionally equivalent to an energy expenditure of 1 kcal per kilogram of body weight per hour of physical activity (34). Thus, total weekly METs initially quantified by the IPAQ in minutes can be converted into mean daily EEE calculated daily as follows:


$$\begin{array}{l} EEE\;daily=\frac{{\left(\frac{MET}{60}\right)}^{*}body\;weight\left(kg\right)}{7} \end{array}$$

The number of MET obtained from the IPAQ (and quantified in minutes) is initially converted into hours by dividing by 60 and then multiplied by body weight to obtain the total energy expenditure, expressed in kcal/week. Finally, the mean daily EEE is obtained by dividing by 7.

### 2.7 Statistical analysis

Statistical analyses were performed using SPSS version 29.0 (SPSS Inc., Chicago, IL). Given the exploratory nature of this study, a formal *a priori* power analysis was not performed, and the sample size was determined based on participant availability and strict adherence to inclusion criteria. The Shapiro-Wilks test was used to verify normality and identify large outliers, via the observation of normality plots. Consequently, continuous variables are presented as means ± standard deviations (SD) for normally distributed data, and as medians and interquartile ranges (IQR) for non-normally distributed variables. Independent samples *t*-tests were conducted to analyze differences between AER and LER in terms of anthropometrics characteristics and EA. Moreover, Cohen's d was calculated as a measure of effect size, and 95% confidence intervals of the mean differences are reported throughout. Additionally, another independent sample *t*-test were conducted to analyze differences between sex for macronutrient and micronutrient intake. Micronutrient intakes were even evaluated against the Italian Dietary Reference Values (LARN) to calculate the proportion of participants meeting nutritional recommendations. Food group consumption frequencies were expressed descriptively and compared alongside the portions suggested by national dietary guidelines.

Pearson correlation analyses were performed to explore associations among EA, MD adherence (MEDI-LITE score), carbohydrate intake (g/kg body weight/day), BMI, and body fat percentage in AER group. A multiple linear regression analysis was performed to identify independent predictors of EA in AER participants. Variables that were significantly correlated with EA in the Pearson correlation analysis were entered simultaneously as predictors in Model 1. In a second step (Model 2), sex was added to evaluate its additional contribution. Multicollinearity was assessed through Variance Inflation Factor (VIF) values.

## 3 Results

### 3.1 Participants characteristics

Out of 84 subjects initially recruited, a total of 67 participants (45 males and 22 females) completed the 7-day food diary. A total of 17 subjects were excluded because they either declined to participate or did not meet the inclusion criteria. The mean age of the participants was 25.5 ± 5.2 years. Overall, participants had a mean BMI of 22.8 ± 2.5 kg/m^2^, and a body fat percentage of 17.7 ± 6.9. The characteristics of the participants are shown in Table 1.

**Table 1:** Participants characteristics.

Parameter	Total (n = 67)(Mean ±SD)	Males (n = 45)(Mean ±SD)	Females (n = 22)(Mean ±SD)
Age (years)	25.5 ± 5.2	25.4 ± 5.1	25.5 ± 5.7
Weight (kg)	69.1 ± 10.1	73.6 ± 8.4	59.8 ± 6.1
Height (cm)	174 ± 8	178 ± 6	165 ± 6
BMI (kg/m^2^)	22.8 ± 2.5	23.3 ± 2.6	21.7 ± 1.6
Waist circumference (cm)	78.0 ± 8.2	81.1 ± 7.6	71.2 ± 4.9
Hip circumference (cm)	94.3 ± 6.2	94.4 ± 6.2	94.2 ± 6.4
WHR	0.83 ± 0.07	0.86 ± 0.05	0.76 ± 0.05
Biceps SKF (mm)	7.2 ± 4.3	6.1 ± 4.0	9.6 ± 3.9
Triceps SKF (mm)	12.7 ± 6.1	10.6 ± 5.2	17.5 ± 5.4
Subscapular SKF (mm)	11.8 ± 4.6	11.9 ± 5.0	11.6 ± 3.5
Suprailiac SKF (mm)	15.5 ± 7.1	15.2 ± 7.6	16.2 ± 6.0
FM (%)	17.7 ± 6.9	14.9 ± 5.1	24.0 ± 6.0
METs/week	3,290.3 ± 1,493.8	3,170.2 ± 1,364.3	3,530.4 ± 1,733.4

BMI, body mass index; WHR, waist-to-hip-ratio; SKF, skinfold; FM, fat mass; METs, metabolic equivalents of task; Data are presented as Mean ± SD.

According to the EI/BMR cut-off values, 45 subjects were classified as AER, while the remaining 22 were classified as LER. No participants were classified as OR. Participants characteristics divided between AER and LER, also differentiated by sex, are shown in Table 2. The data reveal a tendency for volunteers classified as LER to exhibit higher BMI values compared to those classified as AER. Specifically, a significant difference (*p* < 0.05) was observed for BMI, hip circumference, and all skinfold thickness measurements. Moreover, a more pronounced difference (*p* < 0.01) was noted for FM percentage.

**Table 2:** Characteristics of participants, differentiated by “Adequate Energy Reporters” and “Low Energy Reporters” and by sex.

Parameter	Adequate energy reporters (EI/BMR > 1.23) (N = 45)			Low energy reporters (EI/BMR<1.23) (N = 22)			Mean Changes Total (C.I. 95%)	p-Value between Total AER and Total LER	Cohen's d
	Males (N = 32)(Mean ±SD)	Females (N = 13)(Mean ±SD)	Total (N = 45)(Mean ±SD)	Males (N = 13)(Mean ±SD)	Females (N = 9)(Mean ±SD)	Total (N = 22)(Mean ±SD)			
Age (years)	25.4 ± 5.2	23.3 ± 3.6	24.8 ± 4.8	25.5 ± 5.0	28.8 ± 6.8	26.8 ± 5.9	−2.0 (−4.9; 0,9)	0.171	−0.390
Weight (kg)	71.7 ± 7.6	59.5 ± 6.6	68.2 ± 9.2	78.4 ± 8.6	60.2 ± 5.5	71.0 ± 11.7	−2.8 (−8.6; 3.0)	0.340	−0.274
Height (cm)	178.3 ± 6.0	165.3 ± 6.1	174.8 ± 8.4	176.6 ± 5.8	165.5 ± 6.5	172.4 ± 8.1	2.4 (−2.0; 6.8)	0.271	0.293
BMI (kg/m^2^)	22.6 ± 2.4	21.6 ± 1.6	22.3 ± 2.3	25.1 ± 2.3	21.8 ± 1.8	23.9 ± 2.6	−1.6 (−2.9; −0.2)	**0.024** ^*^	−0.658
BMR (kcal/day)	1,757 ± 112	1,358 ± 86	1,642 ± 210	1,847 ± 106	1,389 ± 74	1,660 ± 248	−18 (−143; 106)	0.767	−0.082
Waist circumference (cm)	79.0 ± 5.5	70.9 ± 4.2	76.8 ± 6.3	86.2 ± 9.6	71.7 ± 6.0	80.7 ± 11.0	−3.9 (−8.2; 0.4)	0.072	−0.486
Hip circumference (cm)	92.9 ± 5.6	93.1 ± 5.6	93.0 ± 5.5	98.1 ± 6.3	95.9 ± 7.5	97.3 ± 6.7	−4.3 (−7.7; −0.9)	**0.015** ^*^	−0.730
WHR	0.85 ± 0.1	0.8 ± 0.0	0.83 ± 0.07	0.9 ± 0.0	0.7 ± 0.1	0.83 ± 0.08	0.0 (0.0; 0.0)	0.964	−0.013
Biceps SKF (mm)	4.9 ± 2.2	10.3 ± 4.1	6.4 ± 3.7	9.1 ± 5.7	8.6 ± 3.5	8.9 ± 4.9	−2.5 (−5.0; −0.1)	**0.042** ^*^	−0.618
Triceps SKF (mm)	8.9 ± 4.1	16.9 ± 5.7	11.1 ± 5.8	14.7 ± 5.4	18.5 ± 5.0	16.2 ± 5.5	−5.1 (−8.1; −2.1)	**0.001** ^**^	−0.897
Subscapular SKF (mm)	10.2 ± 3.8	12.0 ± 4.3	10.7 ± 4.0	16.0 ± 5.5	11.0 ± 2.0	14.1 ± 5.1	−3.4 (−5.9; −0.8)	**0.012** ^*^	−0.773
Suprailiac SKF (mm)	12.5 ± 5.4	15.6 ± 6.6	13.3 ± 5.8	22.0 ± 8.2	17.0 ± 5.3	20.1 ± 7.5	−6.8 (−10.5; −3.0)	**0.001** ^**^	−1.052
FM (%)	13.0 ± 4.4	24.2 ± 6.9	16.1 ± 7.2	19.3 ± 4.1	23.6 ± 4.8	21.0 ± 4.89	−4.9 (−7.9; −1.8)	**0.002** ^**^	−0.745
METs/week	3,373.2 ± 1,370.0	3,612.3 ± 1,927.2	3,443.9 ± 1,535.6	2,686.1 ± 1,271.9	3,412.1 ± 1,513.0	2,983.1 ± 1,389.3	460.8 (−295.9; 1217.4)	0.227	0.309

BMI, body mass index; BMR, basal metabolic rate; WHR, waist-to-hip-ratio; SKF, skinfold; FM, fat mass; METs, metabolic equivalents of task. Data are presented as Mean ± SD. ^*^p < 0.05; ^**^p < 0.01 indicate levels of statistical significance. Bold values indicate statistical significance.

### 3.2 Energy and nutrient intake

Mean energy and macronutrient intake of AER participants is shown in Table 3. Protein intake was generally in line with current sport nutrition guidelines. In contrast, the intake of other macronutrients showed higher variability. Specifically, carbohydrate intake did not meet the recommended threshold of 5 g/kg of body weight per day for individuals engaging in moderate level of physical activity, in either male or female participants. Moreover, saturated fat intake appeared to exceed current dietary recommendations, whereas the intake of polyunsaturated fatty acids was below the recommended levels. Fiber intake was not perfectly aligned with the suggested dietary target with a marked difference between males and females. Moreover, when normalized per 1,000 kcal of energy intake, fiber consumption generally fell below recommended levels.

**Table 3:** Mean daily energy and macronutrient intake of participants classified as “Adequate energy reporters”, also differentiated by sex.

Parameter	Adequate energy reporters (EI/BMR > 1.23) (N = 45)				Recommendations
	Males (N = 32) (Mean ±SD)	Females (N = 13) (Mean ±SD)	Total (N = 45) (Mean ±SD)	p-Value between sex	
Energy intake (kcal)	2,649 ± 335	1,960 ± 261	2,450 ± 444	**<** 0.001^***^	
Energy intake (kcal/kg body weight)	37.2 ± 5.5	33.3 ± 5.8	36.1 ± 5.8	**0.039** ^*^	25–35 kcal/kg for people involved in general fitness program40–70 kcal/kg for people involved in moderate level of intense training (4)
Protein (g)	122.1 ± 23.2	84.4 ± 11.3	111.2 ± 26.7	**<** 0.001^***^	
Protein (g/kg body weight)	1.7 ± 0.3	1.4 ± 0.3	1.6 ± 0.3	**0.007** ^**^	0.9–2.0 g/kg (1, 4, 20)
Protein (%)	18.4 ± 2.4	17.3 ± 1.5	18.1 ± 2.3	0.125	
Fat (g)	88.9 ± 15.0	72.2 ± 11.4	84.1 ± 15.9	**<** 0.001^***^	
Fat (%)	30.3 ± 4.2	33.3 ± 3.6	31.2 ± 4.2	**0.037** ^*^	Approximately 30% (reference intake: 20–35% energy) of total energy intake (4, 20)
SFA (%)	11.3 ± 2.3	13.1 ± 2.1	11.8 ± 2.4	**0.014** ^*^	< 10% of total energy intake (1, 20)
MUFA (%)	13.7 ± 2.3	14.2 ± 2.5	13.8 ± 2.3	0.529	
PUFA (%)	4.8 ± 1.0	4.6 ± 1.1	4.8 ± 1.0	0.585	5–10% of total energy intake (20)
Carbohydrates (g)	347.7 ± 59.1	248.0 ± 46.5	318.9 ± 71.7	**<** 0.001^***^	
Carbohydrates (g/kg body weight)	4.9 ± 0.9	4.2 ± 0.9	4.7 ± 0.9	**0.010** ^*^	3–5 g/kg for people involved in general fitness program5–8 g/kg for people involved in moderate exercise program8–12 g/kg for people involved in intense exercise program (1, 4, 20)
Carbohydrates (%)	49.1 ± 4.0	47.3 ± 4.2	48.6 ± 4.1	0.092	45–60% of total energy intake (20)
Sugars (g)	89.5 ± 24.4	80.3 ± 14.2	86.8 ± 22.2	0.107	
Sugars (%)	12.8 ± 2.6	15.6 ± 3.2	13.6 ± 3.7	**0.010** ^*^	< 15% of total energy intake (20)
Fiber (g)	27.6 ± 9.3	18.7 ± 5.0	25.0 ± 9.2	**<** 0.001^***^	>25 g/die (20)
Fiber (g/1,000 kcal)	10.4 ± 3.1	9.6 ± 2.5	10.2 ± 2.9	0.209	12.6–16.7 g/1,000 kcal (20)
Animal proteins (%)	58.4 ± 8.7	60.8 ± 9.5	59.1 ± 8.9	0.212	
Plant proteins (%)	41.1 ± 8.6	38.7 ± 9.3	40.4 ± 8.7	0.203	Try to reach the 40% or even more (20)

SFA, saturated fatty acids; MUFA, monounsaturated fatty acids; PUFA, polyunsaturated fatty acids. Data are presented as Mean ± SD. ^*^p < 0.05; ^**^p < 0.01; ^***^p < 0.001 indicate levels of statistical significance. Bold values indicate statistical significance.

Mean daily micronutrient intake of AER participants is shown in Supplementary Table S1. Available data on the micronutrient content of foods indicate that participants often failed to meet the recommended intakes for some vitamins and minerals, particularly vitamin E, magnesium, and potassium. Notably, female participants appeared more likely to fall short in meeting the recommended intake for additional micronutrients such as vitamin B2 (riboflavin), vitamin B9 (folate), and calcium.

### 3.3 Food groups consumption

Supplementary Table S2 presents the food products recognized in the analysis of food diaries, including the frequency of consumption and weekly quantity for each. The table also shows the suggested quantity and frequency according to the IDG (21). See Supplementary Figures S2–S4 for additional graphical representation, with a comparison between the consumption frequencies observed in the present study and those suggested by the IDG. Additionally, Supplementary Tables S3, S4 shows the 25th, 50th, and 75th percentiles of the food consumption frequency and weekly intake in males and females, respectively.

### 3.4 Adherence to Mediterranean dietary pattern

The MD adherence score, based on the 45 volunteers whose food diaries were considered adequate, was 9.7 ± 2.5 being higher than the MEDI-LITE cut-off value of 8.5 (26). In the study, 68.9% of the volunteers (*n* = 31) were classified as adherent to MD. No volunteer consumed more than 2.5 servings of vegetables per day, and only 13.3% consumed more than 2 servings of fruit per day. Additionally, only 48.9% of the studied population reported regular consumption of olive oil. Table 4 shows the consumption frequencies of food groups, along with the percentage of volunteers consuming different portion sizes according to the MEDI-LITE questionnaire.

**Table 4:** Percentage of volunteers categorized by portion size and food group consumption, according to the MEDI-LITE questionnaire.

Food categories	Frequency of consumption (portion/die or portion/week) and percentage of volunteers (%)		
Fruit (1 portion = 150 g)	< 1 portion/die	1–2 portions/die	>2 portions/die
	**46.7**	40.0	13.3
Vegetables (1 portion = 100 g)	< 1 portion/die	1–2.5 portions/die	>2.5 portions/die
	**44.4**	55.6	0.0
Legumes (1 portion = 70 g)	< 1 portion/week	1–2 portions/week	>2 portions/week
	33.3	22.2	**44.4**
Cereals (1 portion = 130 g)	< 1 portion/die	1–1.5 portions/die	>1.5 portions/die
	2.2	4.4	**93.3**
Fish (1 portion = 100 g)	< 1 portion/week	1–2.5 portions/week	>2.5 portions/week
	15.6	**55.6**	28.9
Meat and cured meat (1 portion = 80 g)	< 1 portion/die	1–1.5 portions/die	>1.5 portions/die
	33.3	**51.1**	15.6
Dairy products (1 portion = 180 g)	< 1 portion/die	1–1.5 portions/die	>1.5 portions/die
	22.2	31.1	**46.7**
Alcohol (1 portion = 12 g)	< 1 AU/die	1–2 AU/die	>2 AU/die
	**88.9**	11.1	0.0
Olive oil	Occasional use	Frequent use	Regular use
	22.2	28.9	**48.9**

The colored background indicates the highest score according to the MEDI-LITE questionnaire, reflecting high adherence to a Mediterranean dietary pattern. Bold indicates the highest percentage of volunteers for each food group.

### 3.5 Energy availability

Table 5 shows the calculated EA, categorized by sex and by AER and LER. Overall, EA appears to be adequate for AER (33.3 ± 7.0), with respect to the threshold 30 kcal/kg FFM that is considered sufficient to maintain body systems. However, it remains below the second recommended threshold of 45 kcal/FFM identified to support healthy physiological functions. Moreover, a significant difference (*p* < 0.001) was observed between AER and LER, as expected. See Supplementary Figure S1 for additional representation.

**Table 5:** Mean energy availability of participants classified as “Adequate energy reporters” and “Low energy reporters”, also differentiated by sex.

Parameter	Adequate energy reporters (EI/BMR > 1.23) (N = 45)			Low energy reporters (EI/BMR<1.23) (N = 22)			Reference values from IOC Consensus Statement	Mean changes total (C.I. 95%)	p-Value Total	Cohen's d
	Males (N = 32)	Females (N = 13)	Total (N = 45)	Males (N = 13)	Females (N = 9)	Total (N = 22)				
EEE (kcal)	566.0 ± 236.0	510.8 ± 276.2	550.0 ± 246.4	499.1 ± 234.7	494.0 ± 228.4	497.0 ± 226.6		57.2 (−65.7; 180.1)	0.399	0.237
EA (kcal/kg FFM)	33.7 ± 6.5	32.3 ± 8.4	33.3 ± 7.0	23.1 ± 6.1	21.8 ± 5.6	22.6 ± 5.8	>30 kcal/kg FFM to avoid perturbation of body systems>45 kcal/kg FFM for healthy physiological function (7)	10.4 (7.2; 13.6)	**< 0.001** ^***^	1.605

EEE, exercise energy expenditure; EA, energy availability. Data are presented as Mean ± SD. ^***^p < 0.001 indicate levels of statistical significance. Bold values indicate statistical significance.

### 3.6 Correlation and regression analyses

Pearson correlation analyses were performed to explore the relationships between on EA, Mediterranean diet adherence, carbohydrate intake, and body composition variables in the AER group (*n* = 45). Results are presented in Supplementary Figure S5. A moderate positive correlation was observed between EA and carbohydrate intake expressed as g/kg body weight (r = 0.491, *p* < 0.001), indicating that participants with higher carbohydrate intake tended to have higher energy availability. Moreover, a moderate negative correlation was observed between EA and BMI (r = −0.326; *p* = 0.031). Carbohydrate intake was also significantly and negatively correlated with both BMI (r = −0.374, *p* = 0.012) and body fat percentage (r = −0.431, *p* = 0.003). The MEDI-LITE score did not show statistically significant correlations with any of the variables examined.

Multiple linear regression analysis (Model 1) revealed that the combination of carbohydrate intake (g/kg body weight) and BMI significantly predicted EA (F(2, 35) = 6.97, *p* = 0.002, Adjusted R^2^ = 0.217). Among the two predictors, carbohydrate intake was the only independent significant predictor of EA (B = 3.15, 95% CI [0.92, 5.39], *p* = 0.007), while BMI did not reach statistical significance after accounting for carbohydrate intake (B = −0.53, 95% CI [−1.43, 0.37], *p* = 0.242). VIF values for both predictors were 1.16, indicating no multicollinearity. The addition of sex in Model 2 did not significantly improve model fit (ΔR^2^ = 0.004, *p* = 0.653), confirming that sex did not independently contribute to the prediction of EA in this sample. Results of regression analysis are presented in Supplementary Table S5.

## 4 Discussion

Optimizing nutritional strategies is a well-recognized determinant of both athletic performance and long-term health of active people (1, 20). Despite the growing number of individuals engaging in regular exercise, dietary habits and energy status of the recreational active population remain largely under-investigated. This study aimed to bridge this gap in the literature by providing a contribution to the field, specifically focusing on the Italian context. Particularly, the primary objective of this study was to evaluate the dietary habits of a group of non-professional athletes and active people by assessing the different food groups consumed weekly and their capacity to cover energy, macronutrient, and micronutrient requirements. Such information could be useful to increase knowledge on the actual food intake of this specific population group and to promote targeted recommendations on how to address potential deficiencies and achieve an optimal diet in line with dietary guidelines. For this reason, adherence to a Mediterranean dietary pattern was also evaluated, as it is considered highly beneficial for the impact on health outcomes and performance in sports people (16, 19).

The first step involved assessing the validity of the dietary records filled in by the volunteers. Consequently, only 45 out of 67 subjects (67.2%) reported plausible energy intakes and thus considered for further analysis. The 33% of records considered inadequate confirm a tendency toward under-reporting, in terms of quantity and quality, of daily food consumption (36). Additionally, we observed that under-reporters presented a higher BMI when compared to adequate reporters; however, it is worth noting that their values remained largely within the normal weight to height range, suggesting that this trend is not linked to overweight status in this cohort. No volunteers were identified as over-reporters.

Considering only AER (*n* = 45), we noticed a tendency to not reach the optimal amount of energy, as well as macro- and micronutrients intake. The ISSN guidelines, along with the Position of the Academy of Nutrition and Dietetics (AND), Dietitians of Canada (DC), and the American College of Sports Medicine (ACSM), alongside the Italian Dietary Reference Values for Nutrients and Energy (LARN), have defined an optimal amount of energy intake for athletes and sports people, as well as for the intake of carbohydrates and proteins (1, 4, 20). Considering the average METs of the volunteers, the participants can be categorized as “active or very active,” reflecting higher energy demands compared to the general population (37). The average EA of the study population was found to be 33.3 ± 7.0 kcal/kg FFM and the proportion of participants meeting adequate EA levels varied considerably depending on the reference threshold applied. If a threshold of 40 kcal/kg FFM/day is considered, as suggested by ISSN guidelines for individuals engaged in moderate-intensity training, only 12 participants (27%) met the recommended energy intake levels, in agreement with previous observations in athletic populations (4, 9, 38). According to the IOC, an EA of 45 kcal/kg FFM is considered optimal for maintaining body weight and supporting physiological functions, while levels below 30 kcal/kg FFM are associated with substantial physiological disruptions (7, 39). In our study, only 2 participants reached the value of 45 kcal/kg FFM; 13 participants (29%) reached 35 kcal/kg FFM, and 33 participants (73%) reached 30 kcal/kg FFM. In contrast, we observed that despite the low EA, participants maintained a healthy body composition and a BMI within the normal range. This discrepancy could potentially be explained by under-reporting of food intake in the dietary records and/or overestimation of physical activity levels as reported in the IPAQ questionnaire. Indeed, it is well established that self-reported physical activity tools tend to systematically overestimate actual activity levels, which would result in an overestimation of EEE and, consequently, an underestimation of EA. This methodological consideration is particularly relevant when interpreting calculated EA values in relation to RED-S risk thresholds, the validity and applicability of which remain a widely debated topic (35, 40). Therefore, conclusions regarding low EA and potential RED-S risk in this sample should be interpreted with caution. Overall, our results reinforce the previously discussed notion that it may be necessary to reassess the validity and applicability of these thresholds in different target groups. Nevertheless, our findings are consistent with those already reported in the literature, which indicate that low EA is commonly observed across various population groups (41–43).

Furthermore, it is important to acknowledge that the body can activate a series of metabolic and hormonal adaptations in response to chronically reduced EA, a pattern described in the literature as “adaptable LEA” (7, 39, 44). These adaptations include a compensatory downregulation of resting metabolic rate, suppression of triiodothyronine (T3) activity at threshold levels of energy availability, and alterations in key hormonal axes including reduced insulin-like growth factor-1 (IGF-1) (6, 39, 45). Such responses represent an energy conservation strategy that may allow individuals to maintain an apparently healthy body composition and metabolic status, at least in the short to medium term, even when EA falls below recommended thresholds. Importantly, the 2023 IOC consensus on REDs emphasizes that LEA exists on a spectrum, and that mild or transient exposure may have minimal impact on long-term health, particularly in the absence of other risk factors (39). This physiological framework, together with the methodological limitations in EA estimation discussed above, provides a more complete explanation for the discrepancy observed between the calculated EA values and the generally healthy body composition of participants.

In terms of macronutrient intake, the importance of carbohydrate as primary fuel source for supporting physical performance is now well established (1, 4, 49). Despite this statement, the literature available to date shows that athletes and sports people often fail to meet the needs in terms of carbohydrate intake (35, 41, 54). According to the ISSN guidelines, the position statement from the AND, DC, and ACSM, and LARN, the recommended range of carbohydrate intake is 5–8 g/kg for people involved in moderate exercise program (1, 4, 20). We observed a tendency to not meet the minimum of 5 g/kg (particularly among female participants), with an average intake of 4.7 ± 0.9 g/kg. Moreover, carbohydrates accounted for roughly 48% of total energy intake, which is near the lower value of the reference intake defined in the LARN (45–60% of total energy intake) (20). Specifically, we observed a particularly low intake of bread, potatoes, rice and whole grains, such as spelt and barley, with respect to IDG (21). Notably, bread consumption was remarkably lower than the suggested intake, while pasta intake remained relatively modest. This behavior is aligned with national trends from the latest Italian national food consumption survey, which shows a general decline in carbohydrate intake in Italy (46). In non-professional athletes, this could represent a significant gap in the daily carbohydrate sources necessary to support training demands. This deficit appears to be partially offset by a preference for other baked products such as savory and sweet snacks. Additionally, subjects reported lower intake of fruit than recommended, which could otherwise contribute to the reduced amount of carbohydrate, as well as other essential nutrients intake. While the total amount of simple sugars was in line with recommendations, just below the upper limit of 15% of energy, the intake of sugary foods, such as ice cream, cakes, and pastries was relatively high. Substituting these with fresh fruit could represent an important dietary target increasing fiber, micronutrient and bioactive intake with potential meaningful health benefits. Furthermore, the amount of dietary fiber consumed is very often lower than the recommended amount and this is particularly critical considering the importance of this nutrient on metabolic and functional aspects that influence physical performance and health.

Overall, we confirmed that the carbohydrate intake requirement appears to be more challenging to meet compared to those for fats and proteins. In this regard, despite recent recommendations and the recognized importance of carbohydrates for athletes, many individuals still believe that carbohydrates are responsible for weight gain and, consequently, a decline in athletic performance (42, 43). Notably, in the present study, higher carbohydrate intake was significantly associated with lower BMI and lower body fat percentage. These data challenge the common perception that carbohydrate intake adversely affects body composition, providing further evidence of its role in supporting a lean body mass in physically active individuals (47). Moreover, Pearson correlation analysis revealed a significant positive association between carbohydrate intake and energy availability, suggesting that adequate carbohydrate consumption is a key determinant of adequate EA. Multiple linear regression analysis confirmed this finding, with carbohydrate intake emerging as the only independent significant predictor of EA, after adjusting for BMI. Conversely, BMI was not an independent predictor of EA. Furthermore, the addition of sex as a covariate did not significantly improve the model fit, indicating that sex did not independently contribute to the prediction of EA in this sample.

As regards protein and fat recommendations, our study seems to suggest that they are generally easily achieved. In fact, participants introduced 1.6 g/kg of body weight of proteins on average. The recommended protein intake for athletes generally ranges from 0.9 to 2.0 g/kg of body weight, leaning more toward the upper limit for strength athletes or individuals engaged in high training volume (1, 4, 20). Our findings align with trends observed in athletic populations, where protein intake is generally well-achieved (13, 48). Moreover, this intake is higher compared to the general Italian adult population, which consistently reports protein consumption levels that easily cover recommendations, although mainly driven by animal sources (46). Also, in this group we observed a generally high intake of animal protein sources, which contributes to the high protein quantity and quality of the diet. However, we also noted a higher intake of red meat and cured meat than recommended. Overall animal proteins accounted for slightly less than two-thirds of the total protein intake, compared to plant proteins. Replacing these foods with legumes may improve overall diet quality by increasing dietary fiber intake and providing additional micronutrients and phytochemicals, while also contributing high-quality carbohydrates and plant protein. Fish, eggs, chicken, and other poultry may represent additional alternatives, providing high-quality protein with generally lower saturated fat content.

In terms of fat intake, the requirements for athletic individuals are similar to or slightly higher than the dietary recommendations for the general population (20–35% of total energy intake) (1, 4, 20). Lipids are an important component, providing essential elements for cell membranes, facilitating the absorption of fat-soluble vitamins, and serving as a source of essential fatty acids (56). Our data suggests that the intake of essential fatty acids is lower than recommended (with PUFA percentages below the 5–10% of total energy intake recommended by LARN), while the intake of SFA is slightly above recommended levels. Dairy products, and in particular cheese, also contributed to SFA intake; however, we noticed a predominant consumption of fresh cheeses rather than aged ones, which differ in serving size according to IDG, likely mitigated the overall saturated fat load. Moreover, participants exhibited a preference for meat over fish consumption. Seafood products in general are one of the primary sources of PUFA, including essential fatty acids (55). A higher intake of nuts could also contribute to the PUFA content of the diet, while we did not observe regular consumption of nuts rich in healthy fat and other critical nutrients for physical activity. In general, for physically active individuals, omega-3 fatty acids, particularly eicosapentaenoic acid (EPA) and docosahexaenoic acid (DHA) present in fish products, are known for their benefits in recovery, performance, and anti-inflammatory properties (52, 53). We also observed a significantly lower intake of Extra virgin olive oil (EVOO) compared to recommendations, with only 48.9% of volunteers consuming it daily. Surprisingly, despite the Mediterranean context, the median consumption frequency did not reach daily intake. This finding requires careful interpretation: while it may partly stem from under-reporting, it also appears to reflect a specific behavior observed in a subset of participants who tend to limit oil consumption, often intentionally omitted from their meals.

In the present study, we observed that individuals often failed to reach the recommended daily intake of some micronutrients Among vitamins, a lower intake with respect to DRV of vitamin B9 and vitamin E was found, which are extremely important for athletes. Furthermore, the intake of some minerals, especially calcium, magnesium, and potassium, was often found lower compared to DRV. Notably, the micronutrient inadequacies observed in our cohort are consistent with broader dietary patterns documented in the general Italian adult population, suggesting that these deficiencies are not exclusive to physically active individuals (50). This can be attributed to the low intake of fruits, vegetables, whole grains, and EVOO, which are key contributors to reaching the recommended daily intake of these nutrients (21). It should also be noted that in the estimation of calcium and other nutrient intakes, the potential contribution from water consumption was not considered, despite water being typically consumed in substantial amounts by physically active individuals.

The level of adherence to MD, derived from the MEDI-LITE questionnaire, suggests that the volunteers had a moderate adherence (mean score 9.7) to the dietary pattern. Considering current literature available, a recent cross-sectional observational study conducted on an Italian soccer team found that most athletes generally adhered to a MD, but with insufficient consumption of fruits, legumes, and fish (13). However, an analysis of the scores for the individual components clearly suggests that targeted modifications to specific dietary habits could further improve adherence to the Mediterranean dietary pattern and increase the overall score. For example, participants tend to consume a high quantity of fresh cheese, as well as meat products and cured meat, while fruit and vegetable consumption was considerably lower than recommended (20, 21). In general, these findings highlight that evaluating diet adequacy requires a comprehensive approach. While adherence scores are valuable for assessing overall diet quality, they should be complemented by quantitative and qualitative assessments to verify that both specific nutrient targets and food group recommendations are well-achieved.

The present study shows some limitations. Firstly, the relatively small sample size of 67 participants (45 when considering only AER), which may not be representative of the broader population of non-professional athletes. This limitation can affect the generalizability of the findings although most of the observations confirm previous findings. Secondly, although the 7-day food diary combined with an interview is currently considered the most accurate method for assessing dietary habits, it relies on self-reported data and remains susceptible to misreporting biases (51). Indeed, a 7-day food diary allows for an accurate estimation of both macro- and micronutrient intake, even though the reliability of nutrients intake, especially for micronutrients, can be affected by the availability of specific data in the food composition databases (23, 24). Furthermore, due to its self-reported nature, the food diary may be susceptible to reporting inaccuracies, especially considering the numerous diaries found to be under-reported. Considering the calculation of EA, there is still no standardized methodology for assessing the various parameters required to calculate it (the number of collection days, methodologies for assessing EI, EEE or FFM) (7). In fact, we did not use gold standard methods for assessing FFM or EEE. Specifically, the estimation of exercise energy expenditure (EEE) based on the IPAQ may not accurately reflect actual physical activity levels, as self-reported questionnaires such as the IPAQ are known to systematically overestimate physical activity levels, which would result in an overestimation of EEE and, consequently, an underestimation of calculated EA. Any inferences regarding RED-S risk thresholds should therefore be interpreted with appropriate caution. Notwithstanding the above, the values we calculated and assessed in the present study are in line with those already reported in the literature and merit further consideration. The integration of the validated MEDI-LITE questionnaire offered an effective tool for assessing adherence to the Mediterranean dietary pattern and the application of EA calculations represents a valuable step toward understanding the balance between energy intake and expenditure in this population. In conclusion, while the methodology could still be improved, the approaches used in this study offer important insights into the dietary habits and energy balance of non-professional athletes. Additionally, the absence of a formal *a priori* power analysis represents a methodological limitation inherent to this study design. As this work was conceived as exploratory and descriptive, with no single primary hypothesis pre-specified, a formal sample size calculation was not feasible.

## 5 Conclusions

The findings underscore a concerning trend of inadequate energy and macronutrient consumption among non-professional athletes, particularly in carbohydrate intake, which is critical for optimal athletic performance. Despite a good adherence to MD, participants sometimes fell short of recommended levels for essential nutrients, including vitamins and minerals. This could be mainly due to the low intake of fruits, vegetables and carbohydrate-rich foods, particularly bread not replaced by other, possibly fiber rich, food sources.

The study emphasizes the need for enhanced nutritional education to promote a more balanced diet that aligns with both performance needs and health recommendations, reducing also misconceptions about carbohydrate consumption and energy intake. Future interventions should focus on improving dietary habits by increasing the consumption of nutrient-dense foods while reducing reliance on high-fat animal products (i.e., red and cured meat, cheese). Addressing these issues may help reduce the risk of Relative Energy Deficiency in Sport and to support the long-term health and performance of athletes. Finally, the conclusions drawn about adherence to MD may be overly simplistic given that dietary patterns are complex and influenced by many factors beyond mere consumption frequency.

## Data Availability

The datasets presented in this article are not readily available because the data supporting the findings of this article are available from the corresponding author, upon reasonable request. Requests to access the datasets should be directed to Patrizia Riso, patrizia.riso@unimi.it.
