# Supplementary material for: Evaluation of food consumption, energy availability and adherence to the Mediterranean dietary pattern in a group of non-professional athletes
Source: Front Nutr. 2026 Jul 24;13:1858754. doi: 10.3389/fnut.2026.1858754 (PMC13449304; doi:10.3389/fnut.2026.1858754)
Supplement: Supplementary file 1 [file Supplementary_file_1.docx]

**Supplementary Figure 1.** Percentage of mean EI, macronutrients and EA as compared to guidelines.

Note: EI: Energy intake; EA: Energy availability. Data are presented as Mean ± SD.
^a^EI with respect to a threshold of 35 Kcal/kg (4)
^b^EI with respect to a threshold of 40 Kcal/kg (4)
^c^Carbohydrate intake with respect to a threshold 5 g/kg (1,4)
^d^Protein intake with respect to a threshold 1.2 g/kg (1,4)
^e^Protein intake with respect to a threshold 1.6 g/kg (1,4)
^f^Fiber intake with respect to a threshold of 25 g/die (20)
^g^EA with respect to a threshold of 30 Kcal/FFM (7)
^h^EA with respect to a threshold of 35 Kcal/FFM
^i^EA with respect to a threshold of 40 Kcal/FFM
^j^EA with respect to a threshold of 45 Kcal/FFM (7)

Supplementary Figure 1 illustrates the extent to which participants, on average, meet the recommended requirements for EI, carbohydrates, proteins, fiber, and EA according to dietary guidelines, considering various thresholds for certain parameters. The different thresholds for the parameters were normalized and expressed as percentages, with 100% representing the target threshold for each parameter. Thus, when a parameter exceeds the 100% threshold, it indicates that the recommended requirement for that parameter has been met or surpassed on average, whereas values below the 100% threshold indicate that the requirement has not been achieved.

**Supplementary Figure 2.** Percentage of median food products consumed weekly as compared to IDG, including males and females participants, with 2500 kcal as a reference (21).

Note: IDG: Italian dietary guidelines; p/die: portions per day; p/week: portions per week. Data are presented as Median with 95% CI.
Milk and yogurt with respect to a standard frequence of 3 p/die (21 p/week)
Cheese with respect to a standard frequence of 3 p/week
Butter and olive oil with respect to a standard frequence of 4 p/die (28 p/week)
Cereals with respect to a standard frequence of 1.5 p/die (10.5 p/week)
Bread with respect to a standard frequence of 4.5 p/die (31.5 p/week)
Potatoes with respect to a standard frequence of 2 p/week
Bread substitutes with respect to a standard frequence of 1 p/week
Breakfast cereals with respect to a standard frequence of 3 p/week
Red meat with respect to a standard frequence of 1 p/week
Poultries with respect to a standard frequence of 3 p/week
Eggs with respect to a standard frequence of 4 p/week
Fish with respect to a standard frequence of 3 p/week
Canned fish with respect to a standard frequence of 1 p/week
Legumes with respect to a standard frequence of 3 p/week
Nuts with respect to a standard frequence of 2.5 p/week
Vegetables with respect to a standard frequence of 3 p/die (21 p/week)
Fruits with respect to a standard frequence of 3 p/die (21 p/week)
Sweet baked products with respect to a standard frequence of 2 p/week

**Supplementary Figure 3.** Percentage of median food products consumed weekly as compared to IDG, including only males participants, with 2500 kcal as a reference (21).

Note: IDG: Italian dietary guidelines; p/die: portions per day; p/week: portions per week. Data are presented as Median with 95% CI.
Milk and yogurt with respect to a standard frequence of 3 p/die (21 p/week)
Cheese with respect to a standard frequence of 3 p/week
Butter and olive oil with respect to a standard frequence of 4 p/die (28 p/week)
Cereals with respect to a standard frequence of 1.5 p/die (10.5 p/week)
Bread with respect to a standard frequence of 4.5 p/die (31.5 p/week)
Potatoes with respect to a standard frequence of 2 p/week
Bread substitutes with respect to a standard frequence of 1 p/week
Breakfast cereals with respect to a standard frequence of 3 p/week
Red meat with respect to a standard frequence of 1 p/week
Poultries with respect to a standard frequence of 3 p/week
Eggs with respect to a standard frequence of 4 p/week
Fish with respect to a standard frequence of 3 p/week
Canned fish with respect to a standard frequence of 1 p/week
Legumes with respect to a standard frequence of 3 p/week
Nuts with respect to a standard frequence of 2.5 p/week
Vegetables with respect to a standard frequence of 3 p/die (21 p/week)
Fruits with respect to a standard frequence of 3 p/die (21 p/week)
Sweet baked products with respect to a standard frequence of 2 p/week

**Supplementary Figure 4.** Percentage of median food products consumed weekly as compared to IDG, including only females participants, with 2000 kcal as a reference (21).

Note: IDG: Italian dietary guidelines; p/die: portions per day; p/week: portions per week. Data are presented as Median with 95% CI.
Milk and yogurt with respect to a standard frequence of 3 p/die (21 p/week)
Cheese with respect to a standard frequence of 3 p/week
Butter and olive oil with respect to a standard frequence of 3 p/die (21 p/week)
Cereals with respect to a standard frequence of 1.5 p/die (10.5 p/week)
Bread with respect to a standard frequence of 3.5 p/die (24.5 p/week)
Potatoes with respect to a standard frequence of 2 p/week
Bread substitutes with respect to a standard frequence of 1 p/week
Breakfast cereals with respect to a standard frequence of 2 p/week
Red meat with respect to a standard frequence of 1 p/week
Poultries with respect to a standard frequence of 2 p/week
Eggs with respect to a standard frequence of 3 p/week
Fish with respect to a standard frequence of 2 p/week
Canned fish with respect to a standard frequence of 1 p/week
Legumes with respect to a standard frequence of 3 p/week
Nuts with respect to a standard frequence of 2 p/week
Vegetables with respect to a standard frequence of 2.5 p/die (17.5 p/week)
Fruits with respect to a standard frequence of 3 p/die (21 p/week)
Sweet baked products with respect to a standard frequence of 2 p/week

Supplementary Figure 2, 3, 4 show how closely participants adhere to the recommended frequency of consumption for various foods or food groups, as outlined by IDG. The total intake in grams for each food group was considered and normalized based on the standard portion size defined by IDG. This allowed us to calculate a normalized frequency per standard portion. Subsequently, we expressed this normalized frequency as a percentage, with 100% representing the recommended frequency for each item according to the IDG. Therefore, the closer the consumption of each food or food group approaches the 100% threshold, the closer it aligns with the recommended frequency for that specific food group.

**Supplementary Figure 5.** Pearson correlation matrix showing the relationships among energy availability, Mediterranean diet score, carbohydrates intake, body mass index and fat mass.

Note: EA: Energy availability; MEDILITE: score derived from the MEDILITE questionnaire; CHO: carbohydrates; BMI: body mass index; FM: fat mass. Numbers within the cells represent Pearson’s correlation coefficients (r). The color bar scale indicates the strength and direction of the associations, ranging from red (strong negative correlation, r = −1.0) to blue (strong positive correlation, r = 1.0). Asterisks denote statistical significance: ∗ p<.05, ∗∗ p<.01 and ***p<.001.
