# Supplementary material for: Evaluation of food consumption, energy availability and adherence to the Mediterranean dietary pattern in a group of non-professional athletes
Source: Front Nutr. 2026 Jul 24;13:1858754. doi: 10.3389/fnut.2026.1858754 (PMC13449304; doi:10.3389/fnut.2026.1858754)
Supplement: Supplementary file 2 [file Supplementary_file_2.docx]

**Supplementary Table 1.** Mean daily micronutrient and mineral intake of participants classified as ‘Adequate energy reporters’, also differentiated by sex.

| **Parameter** | Adequate Energy Reporters (EI/BMR > 1.23) (N = 45) | | | | Dietary reference values LARN | *n* (%) of participants that meet the recommendations | *n* (%) of males that meet the recommendations | *n* (%) of females that meet the recommendations |
| --- | --- | --- | --- | --- | --- | --- | --- | --- |
|  | **Males**  **(N = 32)**  **(Mean ± SD)** | **Females**  **(N = 13)**  **(Mean ± SD)** | **Total**  **(N = 45)**  **(Mean ± SD)** | **p-Value**  **between sex** |  |  |  |  |
| Vit. A (retinol eq.) (mg) | 1287.4 ± 573.9 | 871.3 ± 207.0 | 1167.2 ± 529.3 | **< 0.001***** | 750 males (PRI)  650 females (PRI) | 37 (82%) | 26 (81%) | 11 (85%) |
| Vit. C (ascorbic acid) (mg) | 124.2 ± 66.1 | 90.9 ± 34.6 | 114.6 ± 60.3 | 0.093 | 105 males (PRI)  85 females (PRI) | 29 (64%) | 20 (63%) | 9 (69%) |
| Vit. E (tocopherols, tocotrienols) (mg) | 12.3 ± 3.5 | 8.9 ± 2.7 | 11.3 ± 3.6 | **0.002**** | 13 males (AI)  12 females (AI) | 13 (29%) | 11 (34 %) | 2 (15%) |
| Vit. B1 (thiamine) (mg) | 1.7 ± 0.5 | 1.1 ± 0.4 | 1.5 ± 0.5 | **0.002**** | 0.4 males and females (PRI) | 45 (100%) | 32 (100%) | 13 (100%) |
| Vit. B2 (riboflavin) (mg) | 2.0 ± 0.5 | 1.5 ± 0.3 | 1.8 ± 0.5 | **< 0.001***** | 1.6 males and females (PRI) | 30 (67%) | 26 (81%) | 4 (31%) |
| Vit. B3 (niacin) (mg) | 26.0 ± 7.7 | 18.3 ± 4.6 | 23.8 ± 7.7 | **0.002**** | 18 males and females | 39 (87%) | 31 (97%) | 8 (62%) |
| Vit. B6 (pyridoxine) (mg) | 2.7 ± 0.6 | 1.7 ± 0.4 | 2.4 ± 0.7 | **< 0.001***** | 1.6 males (PRI)  1.4 females (PRI) | 43 (96%) | 32 (100%) | 11 (85%) |
| Vit. B9 (folic acid) (mg) | 396.9 ± 81.1 | 282.1 ± 89.1 | 363.7 ± 97.9 | **< 0.001***** | 330 males and females (PRI) | 30 (67%) | 27 (84%) | 3 (32%) |
| Vit. B12 (cyanocobalamin) (mg) | 6.3 ± 2.9 | 4.8 ± 2.4 | 5.8 ± 2.8 | 0.118 | 4.0 males and females (AI) | 32 (71%) | 24 (75%) | 8 (62%) |
| Calcium (mg) | 975.0 ± 293.8 | 799.6 ± 127.3 | 924.3 ± 267.7 | **0.008**** | 950 males and females (PRI) | 19 (42%) | 17 (53%) | 2 (15%) |
| Iron (mg) | 15.7 ± 3.4 | 11.5 ± 3.2 | 14.5 ± 3.8 | **< 0.001***** | 10 males (PRI)  18 young females (PRI) | 40 (89%) | 31 (97%) | 9 (69%) |
| Magnesium (mg) | 336.9 ± 93.2 | 229.5 ± 51.7 | 305.8 ± 96.3 | **< 0.001***** | 350 males and females (AI) | 12 (27%) | 12 (38%) | 0 (0%) |
| Zinc (mg) | 15.1 ± 2.8 | 10.2 ± 1.1 | 13.7 ± 3.3 | **< 0.001***** | 12 males (PRI)  9 females (PRI) | 40 (89%) | 30 (94%) | 10 (77%) |
| Potassium (mg) | 3617.1 ± 736.9 | 2510.6 ± 392.9 | 3297.4 ± 825.8 | **< 0.001***** | 4500 males and females (SDT) | 4 (9%) | 4 (13%) | 0 (0%) |

*Note: LARN: Reference Intake Levels of Nutrients and Energy for the Italian Population; PRI: Population Reference Intake; AI: Adequate Intake; SDT: Suggested Dietary Target. Data are presented as Mean ± SD. *p < 0.05; **p < 0.01; ***p < 0.001 indicate levels of statistical significance. Bold values indicate statistical significance.*

**Supplementary Table 2.** 25th, 50th, and 75th percentiles of the frequency of consumption and weekly intake of the various food products considered, including both male and female participants.

| **Food groups** | | **Consumption frequencies weekly** | | | **Consumption quantity weekly (g)** | | |  | **Consumption frequencies recommended*** | |
| --- | --- | --- | --- | --- | --- | --- | --- | --- | --- | --- |
|  |  | **25th percentile** | **50^th^ percentile** | **75^th^ percentile** | **25th percentile** | **50^th^ percentile** | **75^th^ percentile** | **Standard portion (g)** | **day** | **week** |
| Dairy | Milk and yogurt | 3.00 | 7.00 | 8.00 | 505.00 | 990.00 | 1540.00 | 125 | 3 |  |
|  | Cheese | 4.00 | 5.00 | 7.00 | 162.50 | 300.00 | 392.50 | 50 for cheese with fat content >25%  100 for cheese with fat content <25% |  | 3 |
|  | Butter | 0.00 | 0.00 | 1.00 | 0.00 | 0.00 | 10.00 | 10 (within the olive oil portion) | 4 (within the olive oil frequency) |  |
| Cereals | Pasta | 3.00 | 4.00 | 6.00 | 325.00 | 520.00 | 705.00 | 80 | 1 ½ |  |
|  | Fresh pasta, stuffed pasta, gnocchi | 0.00 | 1.00 | 1.00 | 0.00 | 80.00 | 250.00 |  |  |  |
|  | Rice | 0.00 | 1.00 | 2.00 | 0.00 | 100.00 | 240.00 |  |  |  |
|  | Other cereals (spelt, barley, others) | 0.00 | 0.00 | 1.50 | 0.00 | 0.00 | 155.00 |  |  |  |
|  | Bread | 3.50 | 6.00 | 9.00 | 300.00 | 420.00 | 714.00 | 50 | 4 ½ |  |
|  | Potatoes | 0.00 | 0.00 | 2.00 | 0.00 | 0.00 | 220.00 | 200 |  | 2 |
|  | Focaccia, pizza, savory pies, flatbreads | 1.00 | 2.00 | 3.00 | 155.00 | 400.00 | 690.00 |  |  |  |
|  | Baked products | 1.00 | 3.00 | 5.00 | 27.50 | 100.00 | 165.50 | 30 |  | 1 |
|  | Breakfast cereals | 0.00 | 1.00 | 4.50 | 0.00 | 30.00 | 165.00 | 30 |  | 3 |
| Protein sources | Beef, lamb, pork | 0.50 | 2.00 | 3.00 | 50.00 | 270.00 | 510.00 | 100 |  | 1 |
|  | Cured meat | 2.00 | 3.00 | 4.50 | 125.00 | 230.00 | 320.00 |  |  |  |
|  | Chicken and other poultry | 0.00 | 2.00 | 3.00 | 0.00 | 300.00 | 460.00 | 100 |  | 3 |
|  | Eggs | 0.00 | 1.00 | 2.00 | 0.00 | 95.00 | 222.50 | 50 |  | 4 |
|  | Fish | 1.00 | 2.00 | 3.00 | 135.00 | 240.00 | 362.50 | 150 |  | 3 |
|  | Canned fish | 0.00 | 1.00 | 2.00 | 0.00 | 50.00 | 135.00 | 50 |  | 1 |
|  | Legumes | 0.00 | 2.00 | 4.00 | 0.00 | 152.00 | 410.00 | 150 |  | 3 |
|  | Protein powder | 0.00 | 0.00 | 0.00 | 0.00 | 0.00 | 0.00 |  |  |  |
| Nuts | Nuts | 0.00 | 1.00 | 6.50 | 0.00 | 20.00 | 105.50 | 30 |  | 2 ½ |
| Vegetables | Vegetables | 5.50 | 7.00 | 10.00 | 620.00 | 1040.00 | 1460.00 | 200 | 3 |  |
| Fruits | Fruits | 4.00 | 6.00 | 13.50 | 505.00 | 930.00 | 1470.00 | 150 | 3 |  |
| Oil and seasoning | Olive oil | 4.00 | 6.00 | 10.00 | 33.50 | 95.00 | 137.50 | 10 (within the butter portion) | 4 (within the butter frequency) |  |
|  | Other seasoning | 0.00 | 2.00 | 3.00 | 0.00 | 35.00 | 130.00 |  |  |  |
| Sugars | Jam | 0.00 | 2.00 | 4.50 | 0.00 | 20.00 | 80.00 |  |  |  |
|  | Fruit juice | 0.00 | 0..0 | 1.00 | 0.00 | 0.00 | 175.00 |  |  |  |
|  | Biscuits | 0.50 | 3.00 | 5.50 | 15.50 | 132.00 | 263.00 | 30-50 |  | 2 |
|  | Cakes and brioches | 0.00 | 2.00 | 5.00 | 0.00 | 140.00 | 332.50 |  |  |  |
|  | Ice cream | 0.00 | 1.00 | 1.00 | 0.00 | 50.00 | 142.50 |  |  |  |
|  | Other sugars | 3.00 | 5.00 | 8.50 | 40.00 | 100.00 | 258.00 |  |  |  |
| Alcohol | Alcoholic beverages | 0.00 | 1.00 | 2.00 | 0.00 | 140.00 | 752.50 |  |  |  |
| Chips | Chips | 0.00 | 0.00 | 0.50 | 0.00 | 0.00 | 7.50 |  |  |  |

Note: *Consumption frequencies refer to IDG, based on a caloric intake level of 2500 kcal.

**Supplementary Table 3.** 25th, 50th, and 75th percentiles of the frequency of consumption and weekly intake of the various food products considered, including only males participants.

| **Food groups** | | **Consumption frequencies weekly** | | | **Consumption quantity weekly (g)** | | |  | **Consumption frequencies recommended*** | |
| --- | --- | --- | --- | --- | --- | --- | --- | --- | --- | --- |
|  |  | **25th percentile** | **50^th^ percentile** | **75^th^ percentile** | **25th percentile** | **50^th^ percentile** | **75^th^ percentile** | **Standard portion (g)** | **day** | **week** |
| Dairy | Milk and yogurt | 3.00 | 6.50 | 8.75 | 483.75 | 932.50 | 1690.00 | 125 | 3 |  |
|  | Cheese | 4.00 | 6.00 | 8.75 | 178.75 | 332.50 | 430.50 | 50 for cheese with fat content >25%  100 for cheese with fat content <25% |  | 3 |
|  | Butter | 0.00 | 0.00 | 1.00 | 0.00 | 0.00 | 14.50 | 10 (within the olive oil portion) | 4 (within the olive oil frequency) |  |
| Cereals | Pasta | 4.00 | 5.00 | 6.00 | 492.50 | 620.00 | 831.25 | 80 | 1 ½ |  |
|  | Fresh pasta, stuffed pasta, gnocchi | 0.00 | 0.00 | 1.00 | 0.00 | 0.00 | 287.50 |  |  |  |
|  | Rice | 0.00 | 1.00 | 2.00 | 0.00 | 115.00 | 300.00 |  |  |  |
|  | Other cereals (spelt, barley, others) | 0.00 | 0.00 | 1.00 | 0.00 | 0.00 | 137.50 |  |  |  |
|  | Bread | 4.25 | 6.50 | 9.00 | 305.00 | 520.00 | 742.50 | 50 | 4 ½ |  |
|  | Potatoes | 0.00 | 0.00 | 1.75 | 0.00 | 0.00 | 250.00 | 200 |  | 2 |
|  | Focaccia, pizza, savory pies, flatbreads | 1.00 | 2.00 | 3.00 | 105.00 | 400.00 | 695.00 |  |  |  |
|  | Baked products | 1.00 | 3.00 | 6.00 | 26.25 | 130.00 | 187.50 | 30 |  | 1 |
|  | Breakfast cereals | 0.00 | 1.00 | 6.00 | 0.00 | 25.00 | 201.25 | 30 |  | 3 |
| Protein sources | Beef, lamb, pork | 0.00 | 2.00 | 3.00 | 0.00 | 305.00 | 549.50 | 100 |  | 1 |
|  | Cured meat | 2.00 | 3.50 | 5.00 | 125.00 | 245.00 | 367.50 |  |  |  |
|  | Chicken and other poultry | 1.00 | 2.00 | 3.75 | 140.00 | 330.00 | 493.25 | 100 |  | 3 |
|  | Eggs | 0.00 | 1.00 | 2.00 | 0.00 | 140.00 | 237.50 | 50 |  | 4 |
|  | Fish | 1.00 | 2.00 | 2.75 | 185.00 | 300.00 | 380.00 | 150 |  | 3 |
|  | Canned fish | 0.00 | 1.00 | 2.00 | 0.00 | 35.00 | 137.50 | 50 |  | 1 |
|  | Legumes | 0.00 | 2.00 | 4.00 | 0.00 | 177.50 | 430.00 | 150 |  | 3 |
|  | Protein powder | 0.00 | 0.00 | 0.00 | 0.00 | 0.00 | 0.00 |  |  |  |
| Nuts | Nuts | 0.00 | 1.50 | 6.75 | 0.00 | 30.00 | 115.00 | 30 |  | 2 ½ |
| Vegetables | Vegetables | 5.00 | 8.00 | 10.00 | 647.50 | 1156.50 | 1920.50 | 200 | 3 |  |
| Fruits | Fruits | 5.00 | 6.50 | 13.50 | 525.00 | 1000.00 | 1497.50 | 150 | 3 |  |
| Oil and seasoning | Olive oil | 4.25 | 8.50 | 10.75 | 40.00 | 105.00 | 157.00 | 10 (within the butter portion) | 4 (within the butter frequency) |  |
|  | Other seasoning | 0.00 | 2.00 | 3.75 | 0.00 | 30.00 | 152.50 |  |  |  |
| Sugars | Jam | 0.00 | 2.00 | 6.50 | 0.00 | 50.00 | 95.00 |  |  |  |
|  | Fruit juice | 0.00 | 0.00 | 0.00 | 0.00 | 0.00 | 0.00 |  |  |  |
|  | Biscuits | 0.00 | 3.00 | 6.00 | 0.00 | 138.00 | 284.00 | 30-50 |  | 2 |
|  | Cakes and brioches | 0.00 | 1.50 | 4.75 | 0.00 | 80.00 | 375.00 |  |  |  |
|  | Ice cream | 0.00 | 0.00 | 1.00 | 0.00 | 25.00 | 138.00 |  |  |  |
|  | Other sugars | 2.00 | 5.00 | 8.00 | 31.25 | 85.00 | 249.00 |  |  |  |
| Alcohol | Alcoholic beverages | 0.00 | 1.00 | 2.75 | 0.00 | 200.00 | 830.00 |  |  |  |
| Chips | Chips | 0.00 | 0.00 | 0.00 | 0.00 | 0.00 | 0.00 |  |  |  |

Note: *Consumption frequencies refer to IDG, based on a caloric intake level of 2500 kcal.

**Supplementary Table 4.** 25th, 50th, and 75th percentiles of the frequency of consumption and weekly intake of the various food products considered, including only females participants.

| **Food groups** | | **Consumption frequencies weekly** | | | **Consumption quantity weekly (g)** | | |  | **Consumption frequencies recommended*** | |
| --- | --- | --- | --- | --- | --- | --- | --- | --- | --- | --- |
|  |  | **25th percentile** | **50^th^ percentile** | **75^th^ percentile** | **25th percentile** | **50^th^ percentile** | **75^th^ percentile** | **Standard portion (g)** | **day** | **week** |
| Dairy | Milk and yogurt | 3.75 | 7.50 | 8.00 | 560.00 | 1002.50 | 1552.50 | 125 | 3 |  |
|  | Cheese | 3.25 | 4.00 | 4.75 | 150.00 | 235.00 | 327.50 | 50 for cheese with fat content >25%  100 for cheese with fat content <25% |  | 3 |
|  | Butter | 0.00 | 0.00 | 0.00 | 0.00 | 0.00 | 0.00 | 10 (within the olive oil portion) | 3 (within the olive oil frequency) |  |
| Cereals | Pasta | 1.25 | 2.50 | 3.75 | 111.25 | 212.50 | 390.00 | 80 | 1 ½ |  |
|  | Fresh pasta, stuffed pasta, gnocchi | 0.00 | 1.00 | 1.00 | 0.00 | 80.00 | 200.00 |  |  |  |
|  | Rice | 0.00 | 1.00 | 1.00 | 0.00 | 85.00 | 123.75 |  |  |  |
|  | Other cereals (spelt, barley, others) | 0.00 | 0.50 | 2.00 | 0.00 | 37.50 | 172.00 |  |  |  |
|  | Bread | 1.50 | 5.00 | 7.75 | 137.50 | 365.00 | 435.00 | 50 | 3 ½ |  |
|  | Potatoes | 0.00 | 0.50 | 2.00 | 0.00 | 49.50 | 195.00 | 200 |  | 2 |
|  | Focaccia, pizza, savory pies, flatbreads | 1.00 | 2.00 | 3.00 | 212.50 | 395.00 | 675.00 |  |  |  |
|  | Baked products | 0.25 | 1.50 | 4.00 | 7.50 | 45.00 | 121.00 | 30 |  | 1 |
|  | Breakfast cereals | 0.00 | 0.50 | 2.75 | 0.00 | 15.00 | 132.50 | 30 |  | 2 |
| Protein sources | Beef, lamb, pork | 1.00 | 2.00 | 2.75 | 120.00 | 240.00 | 382.50 | 100 |  | 1 |
|  | Cured meat | 1.25 | 3.00 | 4.00 | 115.00 | 161.00 | 225.00 |  |  |  |
|  | Chicken and other poultry | 0.00 | 1.00 | 2.00 | 0.00 | 80.00 | 375.00 | 100 |  | 2 |
|  | Eggs | 0.00 | 0.00 | 1.00 | 0.00 | 0.00 | 93.75 | 50 |  | 3 |
|  | Fish | 1.00 | 1.50 | 3.00 | 62.50 | 180.00 | 240.00 | 150 |  | 2 |
|  | Canned fish | 0.00 | 1.00 | 1.75 | 0.00 | 52.50 | 137.00 | 50 |  | 1 |
|  | Legumes | 0.25 | 1.00 | 2.75 | 15.00 | 135.00 | 355.00 | 150 |  | 3 |
|  | Protein powder | 0.00 | 0.00 | 0.00 | 0.00 | 0.00 | 0.00 |  |  |  |
| Nuts | Nuts | 0.00 | 1.00 | 5.75 | 0.00 | 20.00 | 81.75 | 30 |  | 2 |
| Vegetables | Vegetables | 6.00 | 6.50 | 10.25 | 572.50 | 867.50 | 1017.50 | 200 | 2 ½ |  |
| Fruits | Fruits | 3.00 | 6.00 | 13.75 | 320.00 | 782.50 | 1070.00 | 150 | 3 |  |
| Oil and seasoning | Olive oil | 1.25 | 5.50 | 6.75 | 22.50 | 55.50 | 93.75 | 10 (within the butter portion) | 3 (within the butter frequency) |  |
|  | Other seasoning | 0.00 | 2.00 | 3.00 | 0.00 | 65.00 | 128.75 |  |  |  |
| Sugars | Jam | 0.00 | 0.00 | 1.50 | 0.00 | 0.00 | 15.00 |  |  |  |
|  | Fruit juice | 0.00 | 1.00 | 4.50 | 0.00 | 175.00 | 675.00 |  |  |  |
|  | Biscuits | 1.25 | 3.50 | 5.00 | 80.25 | 158.50 | 254.00 | 30-50 |  | 2 |
|  | Cakes and brioches | 1.25 | 2.50 | 5.00 | 57.50 | 160.00 | 291.25 |  |  |  |
|  | Ice cream | 0.00 | 1.00 | 2.00 | 0.00 | 45.00 | 148.75 |  |  |  |
|  | Other sugars | 4.00 | 4.50 | 9.75 | 69.25 | 178.00 | 599.50 |  |  |  |
| Alcohol | Alcoholic beverages | 0.00 | 0.50 | 2.00 | 000 | 50.00 | 545.00 |  |  |  |
| Chips | Chips | 0.00 | 0.00 | 1.00 | 0.00 | 0.00 | 22.50 |  |  |  |

Note: *Consumption frequencies refer to IDG, based on a caloric intake level of 2000 kcal.

**Table 6.** Multiple linear regression analysis with energy availability as the dependent variable in Adequate Energy Reporters.

| **Parameter** | **B** | **SE** | **β** | **p** | **95% CI** |
| --- | --- | --- | --- | --- | --- |
| **Model 1** | | | | | |
| Constant | 30.52 | 12.88 | - | 0.023 | [4.51; 56.53] |
| CHO (g/kg BW) | 3.15 | 1.11 | 0.414 | **0.007** | [0.916; 5.39] |
| BMI (kg/m^2^) | -0.53 | 0.45 | -0.172 | 0.242 | [-1.43; 0.37] |
| *Model fit* | *R = 0.504* | *Adj R^2^ = 0.217* | *F (2,41) = 6.97* | ***p = 0.002*** | *VIF ≤ 1.16* |
| **Model 2** | | | | | |
| Constant | 27.31 | 14.81 | - | 0.073 | [-2.63; 57.24] |
| CHO (g/kg BW) | 3.40 | 1.25 | 0.446 | **0.009** | [0.88; 5.92] |
| BMI (kg/m^2^) | -0.45 | 0.48 | -0.147 | 0.356 | [-1.43; 0.53] |
| Sex | 1.08 | 2.39 | 0.070 | 0.653 | [-3.75; 5.92] |
| *Model fit* | *R = 0.507* | *Adj R^2^ = 0.202* | *F (3,40) = 4.62* | ***p = 0.007*** | *ΔR² = 0.004* |

Note: B: unstandardized regression coefficient; SE: standard error; β: standardized coefficient; 95% CI: 95% confidence interval of B; BW: body weight; VIF: Variance Inflation Factor; ΔR²: change in R-squared when adding sex in Model 2. Energy availability expressed as kcal/kg fat-free mass/day. Sex coded as 0 = male, 1 = female. Model 1: carbohydrate intake and BMI as predictors. Model 2: sex added as additional predictor. Bold values indicate statistical significance.
